# The Impact of Cataract Surgery on Contrast Visual Acuity and Retinal Sensitivity in Patients with Retinitis Pigmentosa

**DOI:** 10.1155/2021/2281834

**Published:** 2021-11-15

**Authors:** Gen Miura, Takayuki Baba, Tomoaki Tatsumi, Hirotaka Yokouchi, Shuichi Yamamoto

**Affiliations:** Department of Ophthalmology and Visual Science, Chiba University Graduate School of Medicine, Chiba, Japan

## Abstract

**Purpose:**

To determine the effects of cataract surgery on contrast visual acuity and retinal sensitivity in patients with retinitis pigmentosa.

**Methods:**

Retinal sensitivity and contrast visual acuity were determined by microperimetry (MAIA) and contrast sensitivity acuity tester (CAT-CP), respectively, before and after cataract surgery. The significance of the correlations between visual acuity, retinal sensitivity, contrast visual acuity, improvements after surgery, and macular structure before and after cataract surgery was determined.

**Results:**

Retinal sensitivity and contrast visual acuity were significantly improved after cataract surgery. The correlations among postoperative visual acuity, postoperative retinal sensitivities, and preoperative ellipsoid zone length were significant. The postoperative retinal sensitivity of the central 10° and the ellipsoid zone length was particularly significantly correlated. Preoperative contrast visual acuity and the amount of improvement and preoperative retinal sensitivity and the amount of improvement were significantly negatively correlated. The contrast visual acuity under both the 100% and 10% photopic and mesopic conditions improved significantly after cataract surgery.

**Conclusions:**

Cataract surgery in retinitis pigmentosa patients with preserved ellipsoid zones significantly improved retinal sensitivity and contrast visual acuity. Cataract surgery can be expected to improve retinal sensitivity and contrast visual acuity under various conditions, even if preoperative visual parameters are low, as long as the ellipsoid zone is preserved.

## 1. Introduction

Cataracts are common complications in all hereditary forms of retinitis pigmentosa (RP) [1]. Cataracts develop at a relatively young age in RP patients [2, 3] and, therefore, impact patients' quality of life due to the loss of visual function. It was recently reported that cataract surgery for RP patients did not appear to be associated with a faster structural progression as measured with OCT [4]. However, it is occasionally difficult to judge whether cataract surgery is beneficial for patients with RP because of problems such as fragile Zinn's zonular fibers, posterior capsular opacification, anterior capsular contraction, macular edema, and difficulty in predicting postoperative visual function. Earlier studies have shown that the findings obtained with the Humphrey Visual Field Analyzer (HFA) and optical coherence tomography (OCT) are important for predicting the visual outcome of cataract surgery in patients with RP [5, 6]. Retinal sensitivity determined by microperimetry and contrast visual acuity (CVA) under different lighting conditions such as photopic and mesopic background are factors of importance in daily life in patients with RP. Even though it is necessary to know the impact of cataract surgery on these parameters, no previous study has performed a detailed analysis and correlation between CVA, retinal sensitivity, and the amount of improvement before and after cataract surgery in RP patients. Thus, the purpose of this study was to evaluate the CVA and retinal sensitivity before and after cataract surgery and to investigate factors that can predict postoperative visual function in patients with RP. The significance of the correlations between best corrected visual acuity, retinal sensitivities by microperimetry, CVA, those improvements after surgery, and preoperative retinal structure were determined to investigate factors affecting CVA and retinal sensitivity after cataract extraction.

## 2. Materials and Methods

This was a retrospective, observational case series of 62 eyes in 62 patients diagnosed with RP. All patients had undergone cataract surgery between January 2009 and January 2018 at Chiba University Hospital. The protocol of this study was approved by the institutional review board of the Chiba University Graduate School of Medicine (No.1781), and the study conformed to the tenets of the Declaration of Helsinki. All patients were informed of the purpose of this study and the procedures to be used, and informed consent was obtained from all patients.

RP was diagnosed based on the presence of a progressive increase in the degree of night blindness, visual field constriction, photophobia, reduced or absent electroretinograms (ERG), and ophthalmoscopic findings, including attenuated retinal vessels, bone-spicule-like pigment clumping, and optic disc pallor. Only patients with bilateral, typical RP were studied. Patients with diabetic retinopathy, uveitis, macular lesions, vitreous macular traction syndrome, macular edema, epiretinal membrane, macular hole, and refractive error (spherical equivalent) greater than ± 6 diopters were also excluded. All eyes underwent phacoemulsification and yellow-colored acrylic foldable intraocular lens implantation surgery through a superior sclerocorneal incision. The hereditary form was predicted clinically based on ocular examination and family history.

The best corrected visual acuity (BCVA), hereditary type, retinal sensitivity, and CVA were evaluated before and 3 months after cataract surgery. Preoperative axial length (AL), ellipsoid zone (EZ) length, and central foveal thickness (CFT) were used to evaluate the relationship between visual function before and after cataract surgery and ocular morphology. Postoperative AL, EZ length, and CFT were not evaluated. The BCVA was measured monocularly using a Japanese standard Landolt ring chart (System Charts SC-2000, Nidek Instruments, Gamagori, Japan) at 5 m, and the decimal visual acuities were converted to logarithm of the minimum angle of resolution (logMAR) units for statistical analyses. The EZ length and CFT were determined from the images obtained with a Spectralis OCT scanner (Heidelberg Engineering, Heidelberg, Germany) from 9 mm scans by two of the authors (GM and HY) in a blinded manner. The image finally used was the average of 100 scans through the fovea. The EZ length was measured based on the definition presented by Staurenghi et al., and the average horizontal and vertical EZ lengths were used for statistical analyses [7]. The distance between the foveal surface of the retina and the inner edge of the retinal pigment epithelium was used as the CFT. All patients were classified into three groups according to EZ status at the fovea: grade 1, EZ not visible; grade 2, EZ abnormal or discontinuous; and grade 3, EZ normal [8].

**Figure 1:**
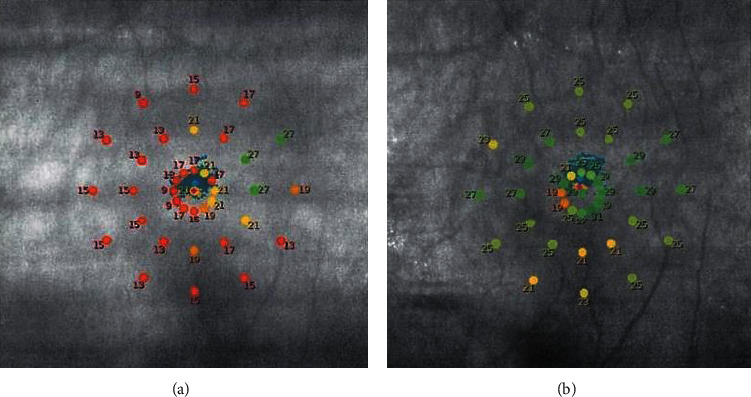
Representative results of macular integrity assessment (MAIA) microperimetry for patients with retinitis pigmentosa before (a) and after (b) cataract surgery. Averaged values of the retinal sensitivities of the central 13 points (2°) and 37 (10°) points were determined.

The retinal sensitivity of the central 2° and 10° was measured by Macular Integrity Assessment (MAIA, CenterVue, Padova, Italy) fundus microperimetry [9]. The average of the predefined central 13 points and 37 points was evaluated as the retinal sensitivities of the central 2° and 10° (Figure 1). The pupils were dilated during the MAIA measurements. We used 0.5% tropicamide and 0.5% phenylephrine (Sandol P; Nitten Pharmaceutical Co., Ltd., Aichi, Japan) to dilate the pupils.

CVA was measured automatedly under two contrast levels of 100% and 10% under photopic (200 cd/m^2^) and mesopic (10 cd/m^2^) conditions with a contrast sensitivity acuity tester (CAT-CP, Neits Instrument, Nagoya, Japan) [10]. The refractive error was corrected for 5 m, and the measurements were performed in a dark room.

We also investigated the incidence of posterior capsular opacification, macular edema, and other complications associated with intraocular lenses within 3 months after cataract surgery.

### 2.1. Statistical Analysis

We determined the significance of the correlations between the preoperative AL, EZ length, CFT, postoperative BCVA, retinal sensitivity, CVA, and improvements in retinal sensitivity and CVA. The significance of the differences in the values before and after surgery was determined by Mann–Whitney tests. Spearman's rank tests were used to determine the significance of any correlations. A *P* value < 0.05 was taken to be statistically significant.

## 3. Results

The demographic baseline characteristics of the patients in this study are shown in Table 1. Cataracts (grades 2 or 3) were present in all cases [11], and they were nuclear, cortical, subcapsular, or a combination of these types. Posterior subcapsular cataract or combination with other cataract types was present in 44 eyes (71.0%). Cortical and nuclear cataracts were present in 10 and 8 eyes, respectively.

The mean preoperative BCVA was 0.45 ± 0.25 logMAR units, and the mean postoperative BCVA was 0.11 ± 0.19 logMAR units. The improvement in postoperative BCVA was statistically significant (*P* < 0.0001). All eyes underwent cataract surgery without any complication. None of the eyes had a decrease in postoperative BCVA, and there were 3 cases in which BCVA did not improve after cataract surgery. None of the eyes developed a posterior capsular opacification that required neodynium:YAG laser capsulotomy. In addition, none of the eyes experienced dislocation of the implanted intraocular lens, macular edema, infection, or prolonged inflammation during the 3-month postoperative observation period. Thirteen of 62 patients were unable to complete the CVA test preoperatively, particularly with low contrast targets under mesopic conditions. The demographics, baseline characteristics, and results of the 49 patients who were able to complete the CVA test are shown in Table 1, as CVA. 61 of 62 subjects had preserved foveal EZ, and only one patient had lost the foveal EZ. The mean preoperative retinal sensitivity of the central 2° was 20.32 ± 4.92 dB, and that of the central 10° was 17.51 ± 6.14 dB. The mean postoperative retinal sensitivity of the central 2° was 23.39 ± 4.02 dB, and that of the central 10° was 19.74 ± 6.23 dB. Both postoperative retinal sensitivities were significantly better than the preoperative retinal sensitivities (*P* < 0.0001).

Correlations between preoperative macular structure and postoperative BCVA and retinal sensitivities are shown in Table 2. The correlations between preoperative EZ length and postoperative BCVA and postoperative retinal sensitivity of central 2° and 10° were significant. Especially, the correlation between preoperative EZ length and postoperative retinal sensitivity of central 10° was the most strong. Preoperative CFT and postoperative retinal sensitivity of central 10° were also correlated significantly.

The postoperative BCVA was weakly correlated with preoperative AL (*r* = −0.298, *P*=0.0184).

The CVA under both the 100% and 10% photopic and mesopic conditions improved significantly after cataract surgery (Table 3). The mean change between the pre- and postoperative CVA under 100% photopic conditions was 0.34 ± 0.25 logMAR units, under 100% mesopic conditions was 0.25 ± 0.22 logMAR units, under 10% photopic conditions was 0.31 ± 0.29 logMAR units, and under 10% mesopic condition was 0.22 ± 0.24 logMAR units. Postoperative CVA was significantly correlated with postoperative BCVA under all conditions (100% photopic, *r* = .510, *P*=0.0001; 100% mesopic, *r* = .627, *P* < 0.0001; 10% photopic, *r* = .284, *P*=0.0478; and 10% mesopic, *r* = .402, *P*=0.0039), and the highest correlation was found under the 100% mesopic condition. The correlations between postoperative CVA, axial length, and macular morphology were not significant.

Our results showed negative correlations among preoperative CVA, preoperative retinal sensitivity, and the amount of improvement in CVA and retinal sensitivity (Table 4). Correlations among EZ length, the amount of improvement of both CVA, and retinal sensitivity were not significant.

## 4. Discussion

In this study, we evaluated the effect of cataract surgery on BCVA in RP patients. We found that the BCVA after cataract surgery was significantly better than the preoperative values, as reported earlier [3, 12]. In addition, the length of the EZ [8, 13] and the CFT [6, 14, 15] in the OCT images was significantly correlated with the postoperative BCVA, as reported earlier. Recently, we reported that cataract surgery can significantly improve the NEI VFQ-25 scores in RP patients and the EZ length can be used to predict the postoperative VFQ scores [16].

Our results also showed that postoperative BCVA was weakly correlated with AL. Usually, a longer AL is associated with progressive worsening of visual function due to chorioretinal atrophy, retinal schisis, and choroidal neovascularization. Several studies have reported a high incidence of macular staphyloma or steeper macular curvature in RP eyes [17, 18]. The authors of those studies assumed that retinal degeneration can not only change the morphology of the neurosensory retina but also alter the shape of the eye, including the macula. However, it remains unclear whether macular staphyloma and changes in the curvature are associated with the progression of retinal degeneration. The reason for the positive correlation between AL and postoperative BCVA found in this study is unclear, but recently, Meinert et al. reported that the macular curvature in RP eyes becomes more concave in eyes with preserved EZ width at >2000 *μ*m [19]. Further investigations on the relationship between ocular morphology and visual function in RP patients are needed.

Both cataract surgery [20] and RP [21] are risk factors for the development of macular edema; however, none of our cases developed macular edema during the 3-month postoperative period. Yoshida et al. reported that none of the RP cases that had macular edema before cataract surgery demonstrated worsening of macular edema after cataract surgery. In addition, none of the patients developed macular edema postoperatively [5]. Our results indicate that cataract surgery in RP patients is not a high-risk factor for developing macular edema in the early postoperative period. However, it should be considered that our conclusion is based on only a 3-month postoperative follow-up, and we did not include patients with preoperative macular edema.

MAIA is a useful method for assessing retinal sensitivities in patients with unstable or eccentric fixation [22]. Battu et al. reported that there was a strong correlation between the retinal structure and sensitivity of the central macula determined by MAIA in patients with RP [9]. The results of our earlier study showed that the annual progression of retinal sensitivity correlated significantly with baseline retinal sensitivity and EZ length in patients with RP [23]. These results indicated that microperimetry findings can be a useful parameter for predicting the progression of RP. The results of another study suggested that microperimetry-determined retinal sensitivity, as opposed to HFA 10-2 program findings, was significantly correlated with the quality of life, and the sensitivity results were significantly correlated with visual function in RP patients [24]. Acton et al. suggested the differences in results between the microperimetry and HFA of patients with RP can be attributed to the different adaptation levels and to the dynamic range of test lights available for the two instruments [25]. We found that postoperative retinal sensitivities of both the central 2° and 10° as evaluated by microperimetry were significantly improved after cataract surgery. There was also a significant correlation between retinal morphology and the retinal sensitivity for the central 10°, which was stronger than that for the central 2°. This finding may be due to the large number of cases in which the length of the foveal EZ was preserved over the central 2°. Our results further showed that the postoperative retinal sensitivity of the central 10° was strongly correlated with the preoperative length of the EZ, suggesting that the length of the EZ is an important factor in predicting postoperative retinal sensitivity. These results indicate that microperimetry is a useful method for evaluating visual function in RP patients after cataract surgery.

Impairment of CVA was confirmed in RP patients [26]. This was important because contrast sensitivity is a good indicator of alterations of the photopic and mesopic visual function in RP patients [27]. Xiao et al. reported that contrast sensitivity, and not the BCVA, showed a linear decrease that was closely associated with the thinning of the outer nerve layer, reduction of the ERG amplitudes, and loss of the cone photoreceptors in rhodopsin-knockout mice [28].

Postoperative CVA was significantly correlated with postoperative BCVA under all conditions. However, CVA was not significantly correlated with the length of the EZ or the CFT. These results are consistent with the findings of an earlier study [26]. The CVA under both the 100% and 10% photopic and mesopic conditions improved significantly after cataract surgery (Table 3). These results indicated that cataract surgery can further improve not only the visual function for high-contrast targets under photopic conditions but also low-contrast targets under mesopic conditions.

Our results showed negative correlations among preoperative CVA, retinal sensitivity, and the amount of improvement (Table 4); however, correlations between EZ length and the amount of improvement of both CVA and retinal sensitivity were not significant. These results indicated that even if preoperative CVA and retinal sensitivity were low, they improved after cataract surgery, regardless of residual EZ length, as long as the EZ was preserved.

This study had several limitations. First, the small number of patients may have weakened statistical analyses. Second, the short follow-up period of 3 months may underlay the low rate of postoperative complications. Third, nearly all of the patients had preserved foveal EZ, and only one patient had lost the foveal EZ. One patient was excluded from the analysis of the CVA because a complete measurement of CVA was not available. Therefore, no conclusions could be drawn regarding the impact of cataract surgery in cases of foveal EZ loss. Fourth, we did not perform genetic testing; thus, the differences in visual function among the different types of RP were not determined.

## 5. Conclusions

In conclusion, cataract surgery significantly improved retinal sensitivity and CVA in RP patients with preserved EZ. The postoperative retinal sensitivity of the central 10° and the length of the EZ were strongly correlated. Cataract surgery has the potential to improve visual function under a variety of contrast and background light conditions, including low-contrast levels and mesopic conditions, in cases with preserved foveal EZ, regardless of the residual EZ length. Our findings support the performance of cataract surgery in RP patients with a preserved EZ. Microperimetry and CVA are useful methods for evaluating the impact of cataract surgery in patients with RP.

## Figures and Tables

**Table 1:** Patient demographics.

	ALL	CVA
Number of eyes	62	49
Age (years)	62.97 ± 12.1	62.4 ± 12.2
Sex (male/female)	34/28	24/25
Hereditary form (AD/AR/sporadic/unknown)	3/7/47/5	3/5/36/5
AL (mm)	23.9 ± 1.40	23.7 ± 1.6
EZ length (*μ*m)	3473.9 ± 2136.6	3800 ± 2190.3
CFT (*μ*m)	258.1 ± 67.3	253.3 ± 70.9
OCT grade (3/2/1)	41/20/1	37/12/0

The EZ evaluated by OCT was classified as grade 1 (absent), grade 2 (abnormal or discontinuous), and grade 3 (normal). The data are displayed as the mean ± SD. AD: autosomal dominant, AR: autosomal recessive, AL: axial length, EZ: ellipsoid zone, CFT: central foveal thickness, OCT: optical coherence tomography.

**Table 2:** Correlations between preoperative retinal constructions and postoperative visual acuity and postoperative retinal sensitivities (*n* = 62).

	Correlation coefficient	*P* value
EZ length vs. BCVA	−0.439	0.0003
EZ length vs. central 2°	0.322	0.01
EZ length vs. central 10°	0.732	<0.0001
CFT vs. central 2°	0.128	0.323
CFT vs. central 10°	0.335	0.0074

EZ: preoperative ellipsoid zone length, BCVA: postoperative best corrected visual acuity, central 2°: postoperative retinal sensitivity of central 2°, central 10°: postoperative retinal sensitivity of central 10°, CFT: preoperative central foveal thickness.

**Table 3:** Changes in BCVA and CVA (*n* = 49).

	Preoperative	Postoperative	*P* value
BCVA	0.43 ± 0.31	0.08 ± 0.16	<0.0001
100% CVA, photopic	0.61 ± 0.25	0.27 ± 0.18	<0.0001
100% CVA, mesopic	0.66 ± 0.20	0.42 ± 0.17	<0.0001
10% CVA, photopic	0.90 ± 0.27	0.60 ± 0.20	<0.0001
10% CVA, mesopic	1.05 ± 0.23	0.83 ± 0.17	<0.0001

BCVA: best corrected visual acuity, CVA: contrast visual acuity.

**Table 4:** Correlations between preoperative contrast visual acuity, preoperative retinal sensitivity, and the amount of improvement in CVA and retinal sensitivity.

	Correlation coefficient	*P* value
100% CVA, photopic	0.730	<0.0001
100% CVA, mesopic	0.675	<0.0001
10% CVA, photopic	0.740	<0.0001
10% CVA, mesopic	0.724	<0.0001
Central 2°	−0.618	<0.0001
Central 10°	−0.265	0.0370

CVA: contrast visual acuity.

## Data Availability

The data used to support the findings of this study are available from the corresponding author upon request.
